# Being overweight worsens the relationship between urinary sodium excretion and albuminuria: the Wakuya study

**DOI:** 10.1038/s41430-023-01327-2

**Published:** 2023-08-16

**Authors:** Kaname Tagawa, Yusuke Tsuru, Katsumi Yokoi, Takanori Aonuma, Junichiro Hashimoto

**Affiliations:** 1 Miyagi University of Education Medical Center, Sendai, Japan; 2 Wakuya National Health Insurance Hospital, Miyagi, Japan; 3 Wakuya Medical and Welfare Center, Miyagi, Japan; 4 Division of Nephrology, Endocrinology, and Vascular Medicine, Department of Medicine, Tohoku University Graduate School of Medicine, Sendai, Japan

**Keywords:** Nutrition, Risk factors

## Abstract

**Background/objectives:**

(Micro)albuminuria (a manifestation of renal microvascular damage) is an independent predictor of mortality risk, even when the urinary albumin/creatinine ratio is ≥ 10 mg/g in the general population. Excessive sodium intake and obesity are strong predictors of cardiovascular disease. However, the effect of obesity on the relationship between sodium intake and albuminuria is not fully understood.

**Subjects/methods:**

The purpose of the present study was to investigate the cross-sectional relationships among dietary sodium intake, obesity, and albuminuria in a general population cohort. Subjects were 928 apparently healthy adults. Body mass index was calculated using the height and body weight. Urinary sodium/creatinine and albumin/creatinine ratios were measured in spot urine samples. Estimated 24-h urinary sodium/creatinine ratio (e24UNa/Cr) was assessed using age, height, body weight, and spot urinary sodium/creatinine ratio.

**Results:**

Both the body mass index and e24UNa/Cr positively correlated with the urinary albumin/creatinine ratio (both, *P* < 0.001), and had a synergistic effect on increasing urinary albumin/creatinine ratio independent of age, sex, mean arterial pressure, and diabetes (interaction *P* = 0.04). When subjects were divided into 6 groups according to the tertiles of e24UNa/Cr and body mass index < (normal-weight) or ≥ 25 (overweight), the prevalence rate of urinary albumin/creatinine ratio ≥ 10 mg/g increased with rising e24UNa/Cr and being overweight (*P* < 0.001).

**Conclusion:**

An increase in body mass index increases the positive association between urinary sodium excretion and (micro)albuminuria in the general population. Excess sodium intake may strengthen cardiovascular risk by increasing (micro)albuminuria, particularly in overweight individuals.

## Introduction

Microalbuminuria (urinary albumin/creatinine ratio [UACR], 30–300 mg/g), a manifestation of renal microvascular damage, is an independent predictor of cardiovascular mortality [1]. Even in normoalbuminuria, a UACR ≥ 10 mg/g predicts all-cause mortality risk in the general population [2]. To mitigate cardiovascular disease (CVD) incidence, UACR is an important factor that can be observed even in apparently healthy individuals.

Obesity is associated with an increased risk of CVD, even for overweight (body mass index [BMI] 25.0–29.9 kg/m^2^) individuals [3]. BMI is a powerful factor for the incidence of type 2 diabetes [4], and individuals with type 2 diabetes have a higher risk of cardiovascular mortality [5]. The risk of type 2 diabetes with increasing BMI is higher in Asians than in whites and blacks [6]. Hence, managing BMI in Asians is a major concern. Additionally, excess sodium intake is a risk factor for CVD [7]. However, the pathophysiological mechanisms underlying the relationship between obesity, sodium intake, and CVD remain unknown.

Obesity is associated with an increased prevalence of microalbuminuria [8]. The association between obesity and prevalence of microalbuminuria is more pronounced in Asians than in other races [8, 9]. Moreover, mild obesity (i.e., overweight) is a risk factor for increased albuminuria in Asians [8]. Excess sodium intake is also a strong factor for increased albuminuria [10]. The relationship between sodium intake and albuminuria varies from individual to individual and is likely to depend on the degree of obesity [11]. The presence of obesity heightens the association between urinary salt excretion and development of microalbuminuria in white and black individuals, but being overweight is not effective [12]. Given the importance of BMI observations for albuminuria in Asians [8, 9], even mild obesity (i.e., overweight) may amplify the effect of sodium intake on albuminuria in Asians. Asians (60%) account for half the world population in 2020, and this proportion has not changed since 1800 [13]. The present study may provide that overweight is an important factor to accelerate the sodium intake-dependent renal microvascular damage in Asians, who represent the majority of the world’s population.

We hypothesized that a combination of excess salt intake and degree of obesity (increased BMI) would increase albuminuria compared with either of these factors. Hence, we sought to investigate the cross-sectional relationships among urinary sodium excretion, BMI, and albuminuria in a Japanese general population cohort.

## Subjects and methods

### Subjects

A total of 928 adults, aged 29–75 years, participated in this study. The present study recruited the general population who underwent a health check-up. All subjects were residents of Wakuya Town or the surrounding areas in the Miyagi Prefecture, Japan. Hypertension was defined as a brachial systolic/diastolic blood pressure ≥140/90 mmHg and/or antihypertensive drug treatment. The diagnosis of type 2 diabetes mellitus was based on antidiabetic drug treatment and/or a medical history of type 2 diabetes mellitus assessed using questionnaires. Hypercholesterolemia was diagnosed based on low-density lipoprotein cholesterol and triglyceride levels of ≥140 mg/dL and ≥150 mg/dL, respectively, and/or cholesterol-lowering drug treatment. Smoking status was evaluated using a questionnaire. All the participants provided written informed consent. The present study was approved by the Institutional Ethics Committee.

### Measurements

#### Hemodynamics

All hemodynamic measurements were performed in a quiet, temperature-controlled environment. Brachial blood pressure and heart rate were measured twice using HEM-9000AI (Omron Healthcare, Kyoto, Japan) in a sitting position after the subjects had rested in a seated position for at least 10 min. The average value was obtained.

#### Anthropometric parameter

The BMI was calculated using body height and weight. Waist circumference at the level of the umbilicus was measured directly using a flexible tape. Normal weight, overweight, and obesity were defined as a BMI < 25, 25–29.9, and ≥ 30 kg/m^2^, respectively. Visceral fat obesity was defined as a waist circumference of at least 85 cm in men and at least 90 cm in women [14].

#### Biochemical parameter

Venous blood samples were obtained to evaluate high-density and low-density lipoprotein, cholesterols, triglyceride, and creatinine levels. Spot urine samples were collected to measure the urinary creatinine, albumin, and sodium levels. Estimated 24-hour urinary sodium/creatinine ratio (e24UNa/Cr) was calculated as follows; 21.98 × (sodium/creatinine in spot urine × estimated 24-h urinary creatinine excretion)^0.392^ [15]. Estimated 24-h urinary creatinine excretion was calculated as −2.04 × age + 14.89 × body weight (kg) + 16.14 × height (cm) − 2244.45 [15]. Normoalbuminuria, microalbuminuria, and macroalbuminuria were defined as UACR < 30, 30–299, and ≥ 300 mg/g, respectively [16]. Estimated glomerular filtration rate (eGFR) was determined using age, sex, and blood creatinine levels with a modified equation specifically for the Japanese population [17]. The presence of kidney dysfunction was defined as an eGFR < 60 ml/min per 1.73 m^2^. The prevalence of CKD was defined as UACR ≥ 30 mg/g and/or eGFR < 60 ml/min per 1.73 m^2^ [18].

### Statistical analysis

All data are presented as mean ± standard deviation (if the distribution was normal), median with interquartile range (if the distribution was skewed), or percentage (for categorical data). An analysis of variance was performed to investigate the following relationships: (a) the univariate relationships between obesity-related parameters (BMI and waist circumference) or e24UNa/Cr and renal parameters (UACR and eGFR), (b) the combined effects of obesity-related parameters and e24UNa/Cr on renal parameters, and (c) subject characteristics divided according to UACR. Multivariate linear analysis was conducted to determine the independent correlates of UACR. UACR ≥ 10 mg/g in the general population is considered an independent predictor of mortality risk [2]. The Kruskal–Wallis test was performed to investigate the combined effects of obesity-related parameters and e24UNa/Cr on the prevalence of UACR ≥ 10 mg/g. Statistical analyses were performed using SPSS (version 21.0; IBM SPSS Japan, Tokyo, Japan). A *P* value of < 0.05 was considered statistically significant.

## Results

### Subject characteristics

The characteristics of the 928 participants are listed in Table 1. The mean age was 56 ± 9 years, and the brachial systolic/diastolic pressure was 125 ± 16/76 ± 11 mm Hg. The mean BMI was 24 ± 3 kg/m^2^. Of the total subjects, 625 (67%) had normal weight, 266 (29%) were overweight, and 37 (4%) were obese. Mean waist circumference was 83 ± 10 cm, and visceral fat obesity was observed in 283 (30%) subjects. The mean e24UNa/Cr was 160 ± 38 mEq/g. The median value and interquartile range of UACR were 5 mg/g and 4–11 mg/g, respectively. Normoalbuminuria, microalbuminuria, and macroalbuminuria were observed in 849 (91%), 70 (8%), and 9 (1%) patients, respectively. The median eGFR was 70 ml/min per 1.73 m^2^ (interquartile range: 64–79 ml/min per 1.73 m^2^), and 181 subjects (20%) had kidney dysfunction. CKD was observed in 208 (22%) patients. From the participants, 362 (39%) had hypertension, 521 (56%) had hypercholesterolemia, and 47 (5%) had diabetes mellitus.

**Table 1:** Characteristics of subjects.

Variables	Total
Clinical measures	
Age, years	56 ± 9
Women, *n* (%)	563 (61)
Height, cm	161 ± 9
Body weight, kg	62 ± 12
Body mass index, kg/m^2^	24 ± 3
Waist circumference, cm	83 ± 10
High-density lipoprotein cholesterol, mg/dL	67 ± 18
Low-density lipoprotein cholesterol, mg/dL	128 ± 31
Triglyceride, mg/dL	116 ± 74
Urinary creatinine, g/L	1.1 ± 0.7
Estimated glomerular filtration rate, mL/min per 1.73 m^2a^	70 (64–79)
Urinary albumin/creatinine ratio, mg/g^a^	5 (4–11)
Normoalbuminuria, *n* (%)	849 (91)
Microalbuminuria, *n* (%)	70 (8)
Macroalbuminuria, *n* (%)	9 (1)
Urinary sodium/creatinine ratio, mEq/g	160 ± 102
Estimated 24-h urinary sodium/creatinine ratio, mEq/g	160 ± 38
Brachial systolic blood pressure, mmHg	125 ± 16
Brachial diastolic blood pressure, mmHg	76 ± 11
Mean arterial pressure, mmHg	95 ± 13
Heart rate, bpm	66 ± 10
Hypertension, *n* (%)	362 (39)
Hypercholesterolemia, *n* (%)	521 (56)
Diabetes mellitus, *n* (%)	47 (5)
Currently smoking, *n* (%)	170 (18)

^a^Data were shown as median (interquartile range).

### Relationships between urinary sodium excretion or BMI and UACR

**Fig. 1:**
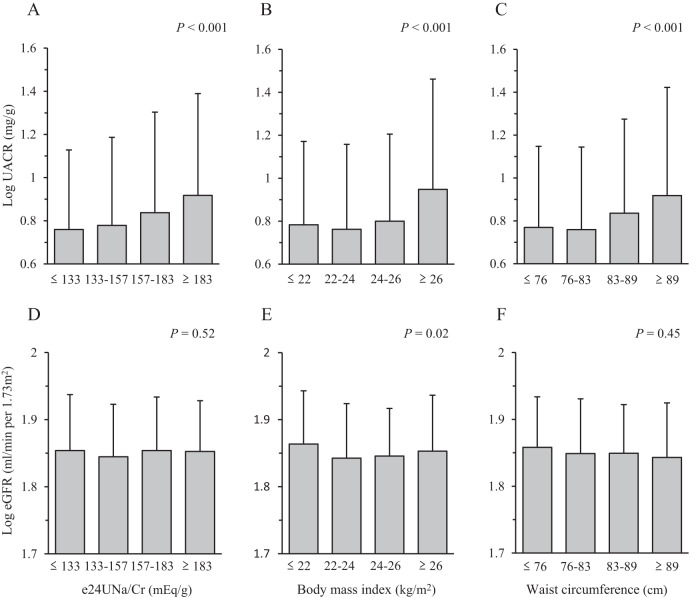
The relationships between estimated 24-h urinary sodium/creatinine ratio, body mass index, or waist circumference and renal parameters (log-transformed urinary albumin/creatinine ratio [UACR] and estimated glomerular filtration rate [eGFR]). The subjects were classified into quartile groups according to the estimated 24-h urinary sodium/creatinine ratio (e24UNa/Cr) (**A** and **D**), body mass index (**B** and **E**), and waist circumference (**C** and **F**). *P* values for trend were assessed by one-way analysis of variance.

Fig. 1 shows the relationship between e24UNa/Cr and obesity-related and renal parameters. There were significant positive associations between e24UNa/Cr, BMI, waist circumference, and log-transformed UACR; namely, UACR increased in a dose-dependent manner with increasing quartiles of e24UNa/Cr, BMI, and waist circumference. The relationships between e24UNa/Cr (*P* = 0.02) or obesity-related parameter (BMI [*P* < 0.001] and waist circumference [*P* < 0.001]) and log-transformed UACR remained significant even after adjustment for age and sex. The association of waist circumference with log-transformed UACR was also significant when separated by sex (Fig. S1). A similar association was observed between BMI and log-transformed eGFR. In contrast, no associations were found between e24UNa/Cr or waist circumference and log-transformed eGFR.

### Combined effects of urinary sodium excretion and BMI on increasing albuminuria

**Fig. 2:**
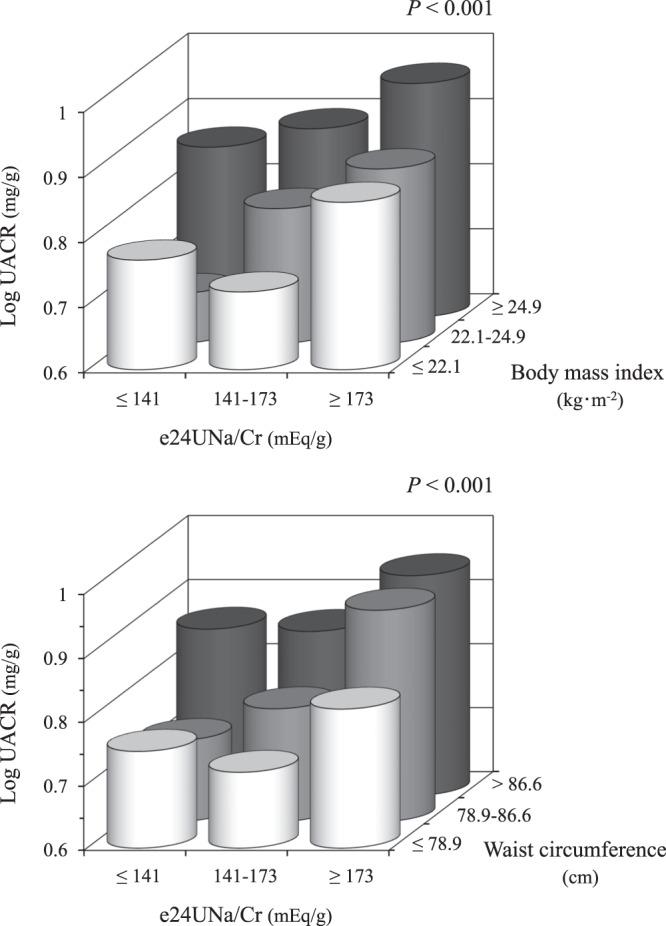
Log-transformed urinary albumin/creatinine ratio (UACR) in subgroups classified according to estimated 24-hour urinary sodium/creatinine ratio (e24UNa/Cr) and body mass index (top) or waist circumference (bottom). *P* values were evaluated using one-way analysis of variance.

Subject characteristics were compared among the tertile groups classified by UACR (Table S1). Subjects in the highest tertile of the UACR were older, and included more women, hypertensive, and diabetic. Of the highest tertile, 247 (80%) were urinary albumin/creatinine ratio ≥10 mg/g. No group differences were observed in the high- and low-density lipoprotein cholesterol levels or in the presence of hypercholesterolemia. When the subjects were divided into nine groups according to tertiles of e24UNa/Cr and BMI, the log-transformed UACR increased with increasing e24UNa/Cr and BMI (Fig. 2, top panel). Similarly, when the subjects were divided according to e24UNa/Cr and waist circumference, an increase in log-transformed UACR was associated with a higher e24UNa/Cr and waist circumference (Fig. 2, bottom panel). Combined effects of e24UNa/Cr and obesity-related parameter on log-transformed UACR were significant even after correction for age and sex (both *P* < 0.001). Combined effects of e24UNa/Cr and waist circumference were also significant when disaggregated by sex (Fig. S2). However, no significant combined effects of e24UNa/Cr, BMI, or waist circumference were observed on the log-transformed eGFR (Fig. S3).

### Synergistic effects of urinary sodium excretion and BMI on increasing albuminuria

Table 2 summarizes the results of multivariate analysis of UACR determinants. When multivariate linear regression analysis was performed with potentially relevant factors, the interaction term between e24UNa/Cr and BMI was found to be a major independent correlate of UACR (*P* = 0.04, model 1). Age, mean arterial pressure, type 2 diabetes mellitus, and BMI were also independent positive correlates of UACR. Meanwhile, sex (women = 0, men = 1) was independently but negatively correlated. When BMI was replaced with waist circumference (model 2), the e24UNa/Cr-related increase in UACR was strikingly greater with increasing waist circumference (*P* = 0.04).

**Table 2:** Independent determinants of log-transformed urinary albumin/creatinine ratio.

Variables	Model 1 (Covariates + body mass index + e24UNa/Cr*body mass index)		Model 2 (Covariates + waist circumference + e24UNa/Cr*waist circumference)	
	Regression coefficient ± SE (×10^−2^)	*P*	Regression coefficient ± SE (×10^−2^)	*P*
Age, years	0.93 ± 0.15	<0.001	0.96 ± 0.16	<0.001
Sex, men/women	−12.69 ± 3.09	<0.001	−13.40 ± 3.14	<0.001
Mean arterial pressure, mmHg	0.56 ± 0.11	<0.001	0.56 ± 0.11	<0.001
Diabetes mellitus, yes/no	26.60 ± 6.24	<0.001	27.02 ± 6.23	<0.001
e24UNa/Cr, mEq/g	0.07 ± 0.04	0.07	0.06 ± 0.04	0.12
Body mass index, kg/m^2^	1.18 ± 0.46	0.01		
e24UNa/Cr*body mass index	0.02 ± 0.01	0.04		
Waist circumference, cm			0.45 ± 0.17	0.01
e24UNa/Cr*waist circumference			0.01 ± 0.004	0.04

e24UNa/Cr, estimated 24-h urinary sodium/creatinine ratio. Covariates were age, sex, high and low-density lipoprotein cholesterols, heart rate, mean arterial pressure, diabetes mellitus, currently smoking, and e24UNa/Cr. R^2^ was 0.12 for models 1 and 2. Both models were *P* < 0.001.

### Distribution of high albuminuria (UACR ≥ 10 mg/g) classified by urinary sodium excretion and BMI

**Fig. 3:**
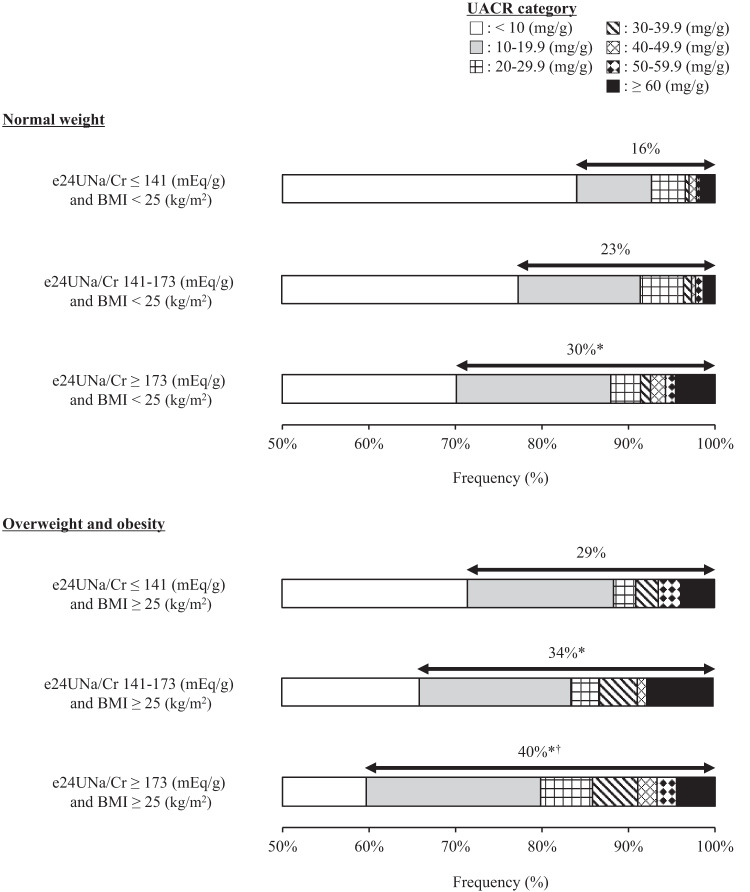
Distribution of urinary albumin/creatinine ratio (UACR) according to tertile of estimated 24-hour urinary sodium/creatinine ratio (e24UNa/Cr) and the presence of at least overweight (body mass index [BMI] ≥ 25 kg/m^2^). Kruskal Wallis test was performed for comparison between groups. *P < 0.05 vs. e24UNa/Cr ≤ 141 mEq/g and BMI < 25 kg/m^2^ group; ^†^*P* < 0.05 vs. e24UNa/Cr 141-173 mEq/g and BMI < 25 kg/m^2^ group.

**Fig. 4:**
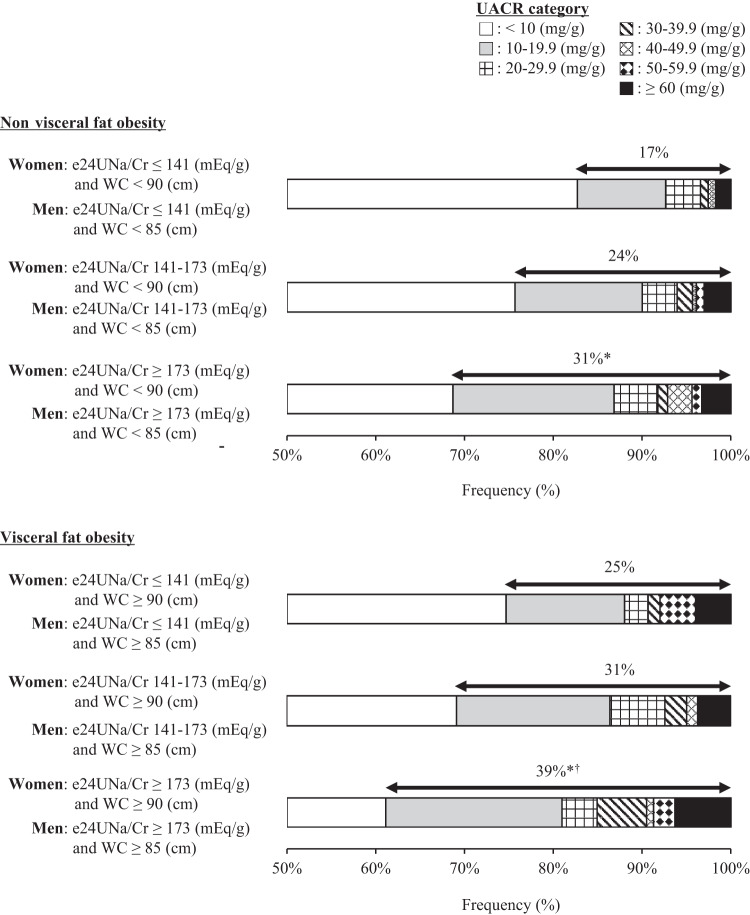
Distribution of urinary albumin/creatinine ratio (UACR) according to tertile of estimated 24-hour urinary sodium/creatinine ratio (e24UNa/Cr) and the presence of visceral fat obesity (waist circumference [WC] of at least 85 cm in men or at least 90 cm in women). Kruskal Wallis test was performed for comparison between groups. *P < 0.05 vs. e24UNa/Cr ≤ 141 mEq/g and WC < 85 cm in men or < 90 cm in women group; ^†^*P* < 0.05 vs. e24UNa/Cr 141–173 mEq/g and WC < 85 cm in men or < 90 cm in women group.

Fig. 3 shows the prevalence rate of UACR ≥ 10 mg/g when the subjects were divided into 6 groups according to the tertile of e24UNa/Cr and normal weight or at least overweight. The prevalence rate of UACR ≥ 10 mg/g increased with increasing e24UNa/Cr and with the presence of overweight status (*P* < 0.001). The prevalence rate of UACR ≥ 10 mg/g was the highest (40%) in the group with highest tertile of e24UNa/Cr and the presence of overweight, and the lowest (16%) in the group with lowest tertile of e24UNa/Cr and normal weight. The result also persisted when the presence of overweight status was replaced with that of visceral fat obesity (*P* < 0.001, Fig. 4).

## Discussion

The present study examined the relationships among urinary sodium excretion, obesity, and albuminuria in the general population of Japan. We found that both e24UNa/Cr and BMI positively correlated with UACR. The e24UNa/Cr-related increase in UACR was strikingly greater with increasing BMI. The overall results did not change when BMI was replaced the waist circumference. Furthermore, the prevalence rate of UACR ≥ 10 mg/g increased with increasing e24UNa/Cr and presence of overweight status or visceral fat obesity. Currently, this is the first study to demonstrate that being overweight magnifies the positive association between urinary sodium excretion and (micro)albuminuria in the Japanese general population.

A positive correlation between urinary sodium excretion and albuminuria is more pronounced with a higher BMI in subjects who are residents of the city of Groningen, Netherlands [11]. The presence of obesity (BMI ≥ 30 kg/m^2^) also heightens the association between urinary sodium excretion and incidence of microalbuminuria in white and black individuals [12]. Findings from the present study are consistent with these previous reports and develop the physiological knowledge known so far in the following points. First, increased urinary sodium excretion and BMI had a synergistic effect on albuminuria in Asians who have a high risk of renal microvascular damage compared to white individuals [19]. Second, when BMI was replaced with waist circumference, urinary sodium excretion-related increases in albuminuria were strikingly greater with increasing waist circumference. Third, 96% of the participants in the current study had normal weight or were overweight. Even a slight increase in BMI within the normal weight to overweight range correlated with an increase in albuminuria. Fourth, the urinary sodium excretion-dependent prevalence rate of high albuminuria (UACR ≥ 10 mg/g) was strengthened by overweight or visceral fat obesity. These findings indicate that even mild obesity (i.e., overweight) strengthens the damage to the renal microvasculature caused by excess salt intake.

Increased glomerular capillary pressure is a major cause of albuminuria [20]. Obese individuals exhibit higher plasma volume and renal plasma flow [21, 22], and obesity-related increases in renal plasma flow lead to increased glomerular capillary pressure [23]. BMI positively correlated with glomerular capillary pressure and albuminuria [20]. Moreover, excessive salt intake increases plasma volume and albuminuria [10, 24]. Considering these reports and new findings from the current study, combined increases in BMI and excess salt intake may increase renal microvasculature damage potentially by increasing the glomerular capillary pressure caused by higher renal blood flow.

The presence of obesity heightens the correlation between urinary sodium excretion and development of microalbuminuria in white and black individuals; however, being overweight does not have a heightening effect [12]. The present study found that the association between urinary sodium excretion and albuminuria was magnified with increasing BMI in the general Japanese population, where many subjects were normal or overweight. The association was not altered when BMI was replaced with waist circumference, suggesting that visceral fat obesity and mild obesity (i.e., overweight) adversely affect the association between urinary sodium excretion and albuminuria in the general Japanese population. Asians have a higher incidence of microalbuminuria or macroalbuminuria than whites [19]. The close intertwinement of overweight and excessive salt intake may partially explain the high incidence of microalbuminuria in Asians. Furthermore, the present study observed that the prevalence of UACR ≥ 10 mg/g increased with increasing urinary sodium excretion and with the presence of overweight status or visceral fat obesity. UACR ≥ 10 mg/g is a strong predictor of CVD mortality in the general population [2]. The present study provides valuable evidence that combined excess salt intake and mild obesity increases CVD risk, referable to increased albuminuria.

Visceral fat obesity is associated with a greater risk for microalbuminuria, even in individuals with normal weight [25], suggesting that both BMI and waist circumference should be controlled to prevent damaging the renal microvasculature. This study revealed for the first time that visceral fat obesity strengthens the association between urinary sodium excretion and albuminuria. Thus, visceral fat obesity may magnify the increased CVD risk owing to excessive salt intake. This novel finding provides additional evidence of the importance of waist circumference in preventing renal microvascular damage.

The worldwide average salt intake is known to be about 10 g (170 mEq)/d [26]. Overweight is defined as a BMI 25–29.9 kg/m^2^, and visceral fat obesity is defined as a waist circumference of at least 85 cm in men and at least 90 cm in women [14]. As shown in Fig. 1, UACR value was the highest in highest quartiles of e24UNa/Cr (≥ 183 mEq/g), BMI (≥ 26 kg/m^2^), and waist circumference (≥ 89 cm). Therefore, the individuals with the worldwide average salt intake and the presence of overweight or visceral fat obesity had the higher levels of albuminuria in the present study. In addition, the worldwide average salt intake (e24UNa/Cr ≥ 173 mEq/g) and the presence of practically overweight (BMI ≥ 24.9 kg/m^2^) or practically visceral fat obesity (waist circumference ≥ 86.6 cm) had combined effects on increasing albuminuria (Fig. 2). Taken together, this study may provide clinically important implications.

The current study had some limitations. First, the cross-sectional nature of the study precludes definitive cause-and-effect conclusions. The causal relationships between sodium intake, obesity, and renal microvascular damage need to be tested further in prospective studies. Second, this study used e24UNa/Cr from spot urine samples. A 24-h urine collection is the most accurate method for assessing sodium intake, but collecting a complete and accurate 24-h urine sample is difficult. The 24-h urinary sodium/creatinine ratio estimated from spot urine is a practical alternative for general medical facilities [15]. Third, since the subjects of this study cohort were the general Japanese population, many of the subjects were non-obese (96%). Whether the same results as this study were obtained in obese subjects and whether there are racial differences needs further clarification. Fourth, confounding factors such as protein consumption, salt intake, physical activity, and muscle mass, which were not measured in this study, may influence the associations between sodium excretion, obesity, and renal microvascular damage. Further studies will be necessary to conduct a detailed analysis that excludes these effects.

In summary, increases in BMI and waist circumference strengthened the positive association between urinary sodium excretion and (micro)albuminuria. Moreover, the presence of overweight status and visceral fat obesity magnified the relationship between urinary sodium excretion and the prevalence of UACR ≥ 10 mg/g, an independent predictor of mortality risk in the general population [2]. Excess salt intake could potentially have a pernicious effect on CVD risk through renal microvascular damage, especially in overweight individuals.

### Supplementary information

Supplemental Digital Content

## Data Availability

The datasets generated during and/or analyzed during the current study are available from the corresponding author on reasonable request.
