# Supplementary material for: Being overweight worsens the relationship between urinary sodium excretion and albuminuria: the Wakuya study
Source: Eur J Clin Nutr. 2023 Aug 16;77(11):1044–50. doi: 10.1038/s41430-023-01327-2 (PMC10630129; doi:10.1038/s41430-023-01327-2)
Supplement: Supplementary file 1 — Supplemental Digital Content [file 41430_2023_1327_MOESM1_ESM.pdf]

Being overweight worsens the relationship between urinary sodium excretion and albuminuria: the Wakuya study

Kaname Tagawa[1], Yusuke Tsuru[2], Katsumi Yokoi[2], Takanori Aonuma[3], and Junichiro Hashimoto[1, 4]

Affiliations:

[1]Miyagi University of Education Medical Center, Sendai, Japan; [2]Wakuya National Health Insurance Hospital, Miyagi, Japan; [3]Wakuya Medical and Welfare Center, Miyagi, Japan; [4]Division of Nephrology, Endocrinology, and Vascular Medicine, Department of Medicine, Tohoku University Graduate School of Medicine, Sendai, Japan

Running head: Sodium, obesity and albuminuria

Corresponding author:

Junichiro Hashimoto, MD, PhD

Medical Center, Miyagi University of Education,

149 Aoba, Aramaki, Aoba-ku, Sendai, Miyagi 980-0845, Japan.

Email: hashimoto@med.tohoku.ac.jp

Table S1. Subject characteristics according to urinary albumin/creatinine ratio

Variables	Tertiles of urinary albumin/creatinine ratio			
	Lowest n = 309	Middle n = 309	Highest n = 310	*P*
Age, years	54 ± 8	56 ± 9	59 ± 9	< 0.001
Women, n (%)	147 (48)	214 (69)	202 (65)	< 0.001
Body weight, kg/m^2	63 ± 11	60 ± 12	62 ± 12	< 0.001
High-density lipoprotein cholesterol, mg/dL	67 ± 18	67 ± 17	66 ± 19	0.73
Low-density lipoprotein cholesterol, mg/dL	129 ± 31	126 ± 32	128 ± 31	0.43
Triglyceride, mg/dL	121 ± 92	107 ± 61	120 ± 64	0.03
Brachial systolic blood pressure, mmHg	122 ± 15	123 ± 16	130 ± 17	< 0.001
Brachial diastolic blood pressure, mmHg	74 ± 11	75 ± 11	78 ± 12	0.001
Mean arterial pressure, mmHg	92 ± 12	94 ± 13	97 ± 13	< 0.001
Heart rate, bpm	65 ± 10	66 ± 9	67 ± 10	0.01
Urinary albumin/creatinine ratio, mg/g*	3 (2–4)	5 (5–6)	14 (11–26)	< 0.001
Hypertension, n (%)	77 (25)	105 (34)	180 (58)	< 0.001
Diabetes mellitus, n (%)	7 (2)	9 (3)	31 (10)	< 0.001
Hypercholesterolemia, n (%)	168 (54)	164 (53)	189 (61)	0.11
Currently smoking, n (%)	76 (25)	44 (14)	50 (16)	0.002

*Data were shown as median (interquartile range).

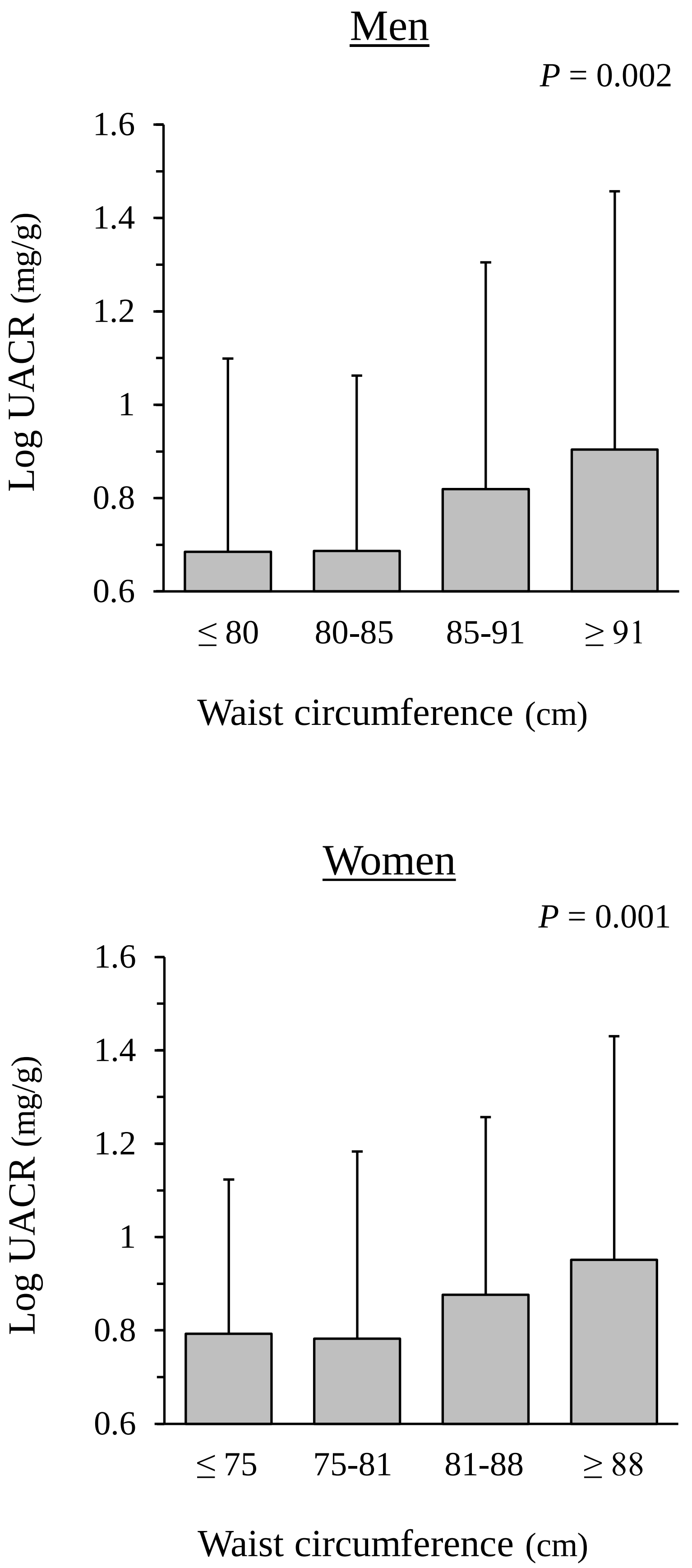

Figure S1. The relationship between waist circumference and log-transformed urinary albumin/creatinine ratio (UACR) by sex.

Men

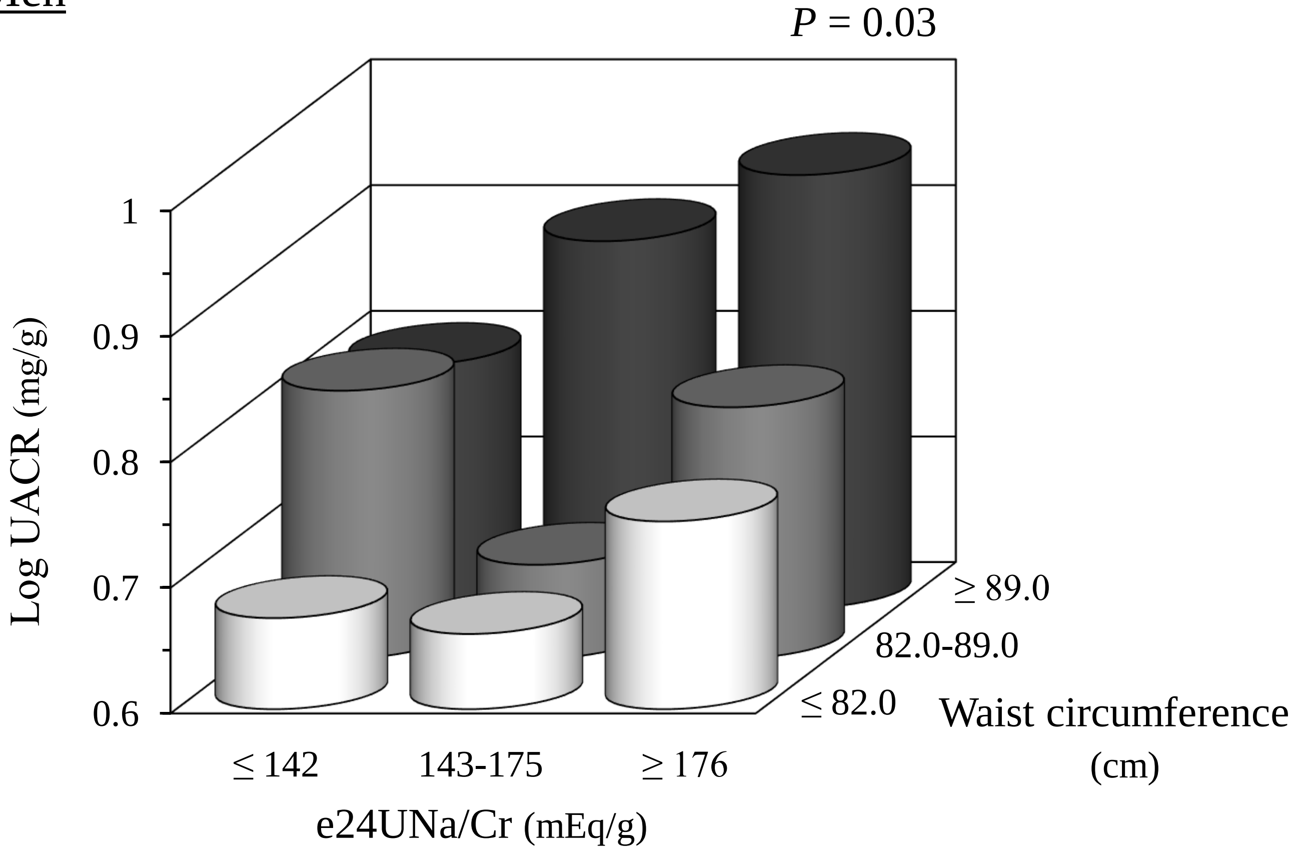

Women

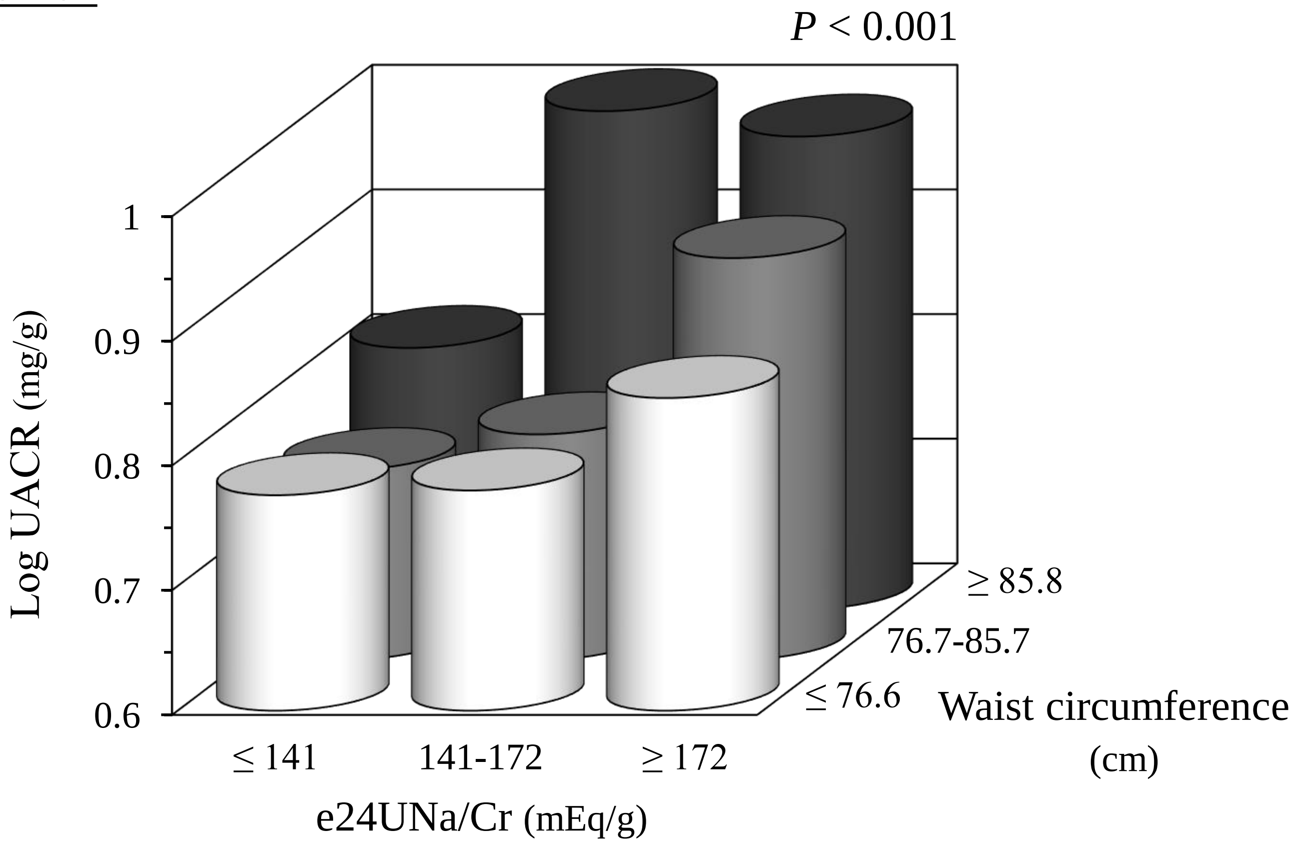

Figure S2. Log-transformed urinary albumin/creatinine ratio (UACR) in subgroups classified according to estimated 24-hour urinary sodium/creatinine ratio (e24UNa/Cr) and waist circumference by sex.

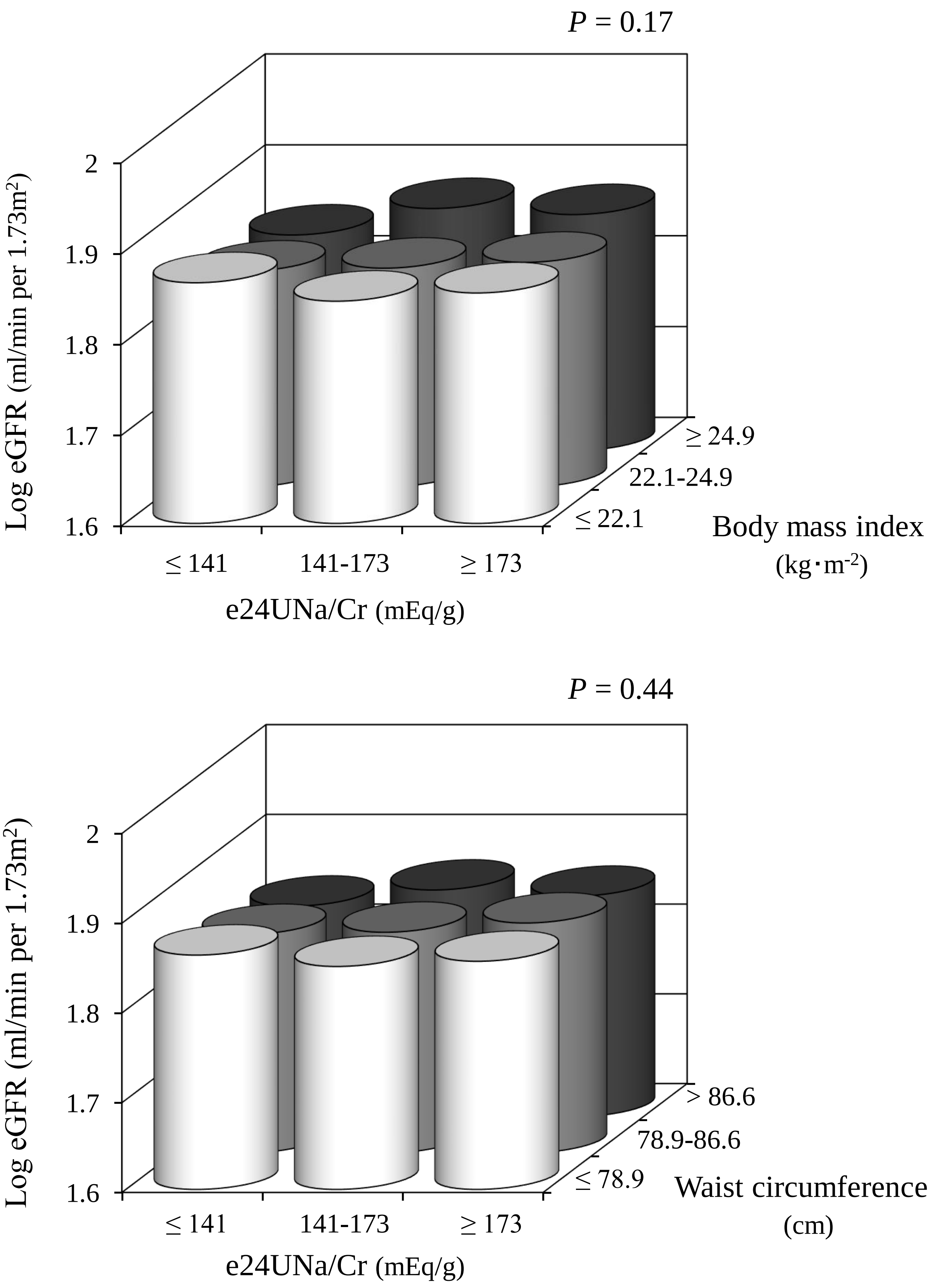

Figure S3. Log-transformed estimated glomerular filtration rate (eGFR) in subgroups classified according to estimated 24-hour urinary sodium/creatinine ratio (e24UNa/Cr) and body mass index (top) or waist circumference (bottom).
